# Flat Panel Light Source with Lateral Gate Structure Based on SiC Nanowire Field Emitters

**DOI:** 10.1038/srep10976

**Published:** 2015-06-04

**Authors:** Meng-Jey Youh, Chun-Lung Tseng, Meng-Han Jhuang, Sheng-Cheng Chiu, Li-Hu Huang, Jyun-An Gong, Yuan-Yao Li

**Affiliations:** 1 Department of Information Technology, Hsing Wu University, New Taipei City 244, Taiwan, R.O.C.; 2 Wafer Process Engineering Department, Epistar Corporation, Tainan 744, Taiwan, R.O.C; 3 Graduate Institute of Opto-Mechatronics, National Chung Cheng University, Chia-Yi 621, Taiwan, R.O.C; 4 Product Technology Department, AU Optronics Corporation, Taoyuan 325, Taiwan, R.O.C; 5 Advanced Institute of Manufacturing with High-Tech Innovations, National Chung Cheng University, Chia-Yi 62102, Taiwan, R.O.C; 6 Department of Chemical Engineering, National Chung Cheng University, Chia-Yi 621, Taiwan, R.O.C

## Abstract

A field-emission light source with high luminance, excellent luminance uniformity, and tunable luminance characteristics with a novel lateral-gate structure is demonstrated. The lateral-gate triode structure comprises SiC nanowire emitters on a Ag cathode electrode and a pair of Ag gate electrodes placed laterally on both sides of the cathode. The simple and cost-effective screen printing technique is employed to pattern the lateral-gates and cathode structure on soda lime glass. The area coverage of the screen-printed cathode and gates on the glass substrate (area: 6 × 8 cm^2^) is in the range of 2.04% – 4.74% depending on the set of cathode-gate electrodes on the substrate. The lateral-gate structure with its small area coverage exhibits a two-dimensional luminance pattern with high brightness and good luminance uniformity. A maximum luminance of 10952 cd/cm^2^ and a luminance uniformity of >90% can be achieved with a gate voltage of 500 V and an anode voltage of 4000 V, with an anode current of 1.44 mA and current leakage to the gate from the cathode of about 10%.

Lighting accounts for a significant part (20%) of all electrical energy consumed worldwide. Next-generation light sources have thus been extensively studied to reduce energy consumption and lower environmental impact. Flat-panel lighting emits light over a large area, such as an entire wall. Unlike incandescent lights (point sources) and fluorescent lights (tube sources), flat-panel lighting does not require elaborate interconnections and light scattering technology to deliver even illumination. Some practical advantages of this technology include: (1) glare reduction (the total lumen output is distributed over a large area), (2) thin lighting modules (likened to a sheet of glass), and (3) long operating (service) life. In general, flat-panel lighting can be used anywhere that conventional lamps are used, such as for ceiling lighting, wall lighting, and both indoor and outdoor decorative lighting. Flat-panel lighting has been applied as a backlight source for high-resolution medical displays, air traffic control displays, industrial display units, and signage^1^,^2^,^3^,^4^.

Field-emission light sources, an emissive type of flat panel technology, employs cold field emitters in place of thermal cathodes as electron sources. Each cold emitter is a nano-scale sharp tip that emits electrons upon the application of a high electrical field. The electrons travel through a vacuum and bombard the phosphor coatings applied to an anode and the faceplate glass that forms the screen. The electrical field emitter is one of the most essential components in a field emission device. Emitters require a low effective work function, high geometrical aspect ratio, and good thermal and chemical stability^5^,^6^,^7^. A variety of one-dimensional (1D) nanomaterials have been studied as novel emitters, including 1D carbon nanomaterials^8^,^9^,^10^,^11^, 1D nano-metal oxide^12^,^13^,^14^,^15^, and 1D nano-metal materials^12^,^16^,^17^,^18^. However, from a practical point of view, the in situ growth of emitters at low temperature (<450 °C) on a desired substrate or the production of emitters in powder form are required for industrial applications. Emitters in powder form can be used in gram-scale quantities for device fabrication using techniques such as screen-print patterning. Carbon nanotubes (CNTs) are a promising material for such purposes, and have thus been extensively studied for field emission applications^19^,^20^.

Silicon carbide (SiC) is a semiconducting material that possesses high thermal stability, good chemical resistivity, good mechanical strength, and unique optical properties. Many 1D SiC nanostructures such as nanorods^21^,^22^,^23^, nanowires^24^,^25^, nanotubes^26^,^27^, nanobelts^28^,^29^, and nanocables^30^,^31^,^32^ have been synthesized. The field emission properties of such structures have been studied^33^,^34^. Compared to CNT emitters, SiC nanoemitters have better thermal stability and chemical resistance, which are beneficial for the device fabrication process and improve device stability and lifetime. For example, in a field-emission lighting test, CNTs can become damaged or burn out due to the heat generated by high electrical current density. SiC has better thermal stability and can thus withstand higher thermal shock. In our previous studies, large quantities of SiC nanowires were produced via a modified chemical vapor deposition (CVD) method^24^ and their field emission characteristics were studied using a field emission device with a parallel-plate diode structure^8^. It was found that SiC nanowires have great potential for field emission applications.

The present study investigates the feasibility of field-emission lighting that uses SiC nanowires as emitters. A field emission device with a lateral-gate triode structure was fabricated using a cost-effective screen printing process. Systematic field emission experiments were carried out to find optimal operating conditions for high luminance and good uniformity.

## Experimental details

SiC nanowires were produced via a combination of CVD and the CNT confined reaction method^24^. Briefly, Fe-Ni catalytic nanoparticles on Si-SiO_2_ core-shell powder were synthesized with a thermal treatment at 550 °C in air. CVD was conducted using C_2_H_2_ as the carbon source in the presence of the catalyst at 650 °C for 1 hr for the synthesis of CNTs, which was followed by increasing the temperature to 1300 °C under an argon atmosphere for the synthesis of SiC nanowires. The as-produced powder was placed in a 4N NaOH solution at 60 °C for 5 hr for removing the catalyst from the SiC nanowire powder. A screen-printable SiC nanowire paste comprised SiC nanowires and an organic vehicle. The organic vehicle was a mixture of ethyl cellulose (Aldrich), α-terpineol (Aldrich), and diethylene glycol monobutyl ether (Aldrich) with a weight ratio of 3:1:1. The organic vehicle was used to disperse the SiC nanowires and control the viscosity of the paste. The purified SiC nanowire powder (0.5 g) and the organic vehicle (15 g) were well mixed by a mixing/degassing machine (Thinky ARE-250) to become a printable paste.

The structures of lateral-gate/cathode electrodes were printed on soda lime glass (area: 6 × 8 cm^2^) by a screen printer (Hotshine, model 2020HIC) using Ag paste (DuPont, FL-5773B) and the prepared SiC nanowire paste. A baking process at 100 °C for 10 min and then a firing process at 400 °C for 60 min were conducted after the printing process in order to remove the solvent and organic binders. ZnS green phosphors with a particle size of 1–4 μm were screen-printed on indium tin oxide (ITO) glass (area: 5 × 5 cm^2^) as the anode. The gap between two parallel panels (anode and gate-cathode panels) was 1 cm.

**Figure 1:**
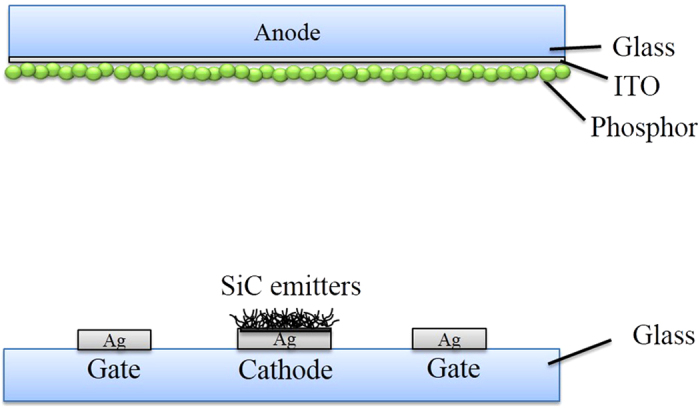
Schematic diagram of lateral-gate triode structure of field emission device.

The morphology and crystalline structure of the SiC nanowires were examined by field-emission scanning electron microscopy (FE-SEM, HITACHI S-4800), high-resolution transmission electron microscopy (HR-TEM, Philips Tecnai F20), and X-ray diffraction (XRD, RIGAKU Miniflex) using CuKα radiation. The field emission characteristics were examined in a vacuum chamber at a pressure of 6 × 10^−6^ Torr. The emission current was monitored with a source meter (Keithley 2410) while the brightness and luminescence uniformity were measured by a luminance colorimeter (BM-7A, Topcon). The gate and cathode were direct-current (DC) biased ranging from 200 to 500 V, while the anode and cathode were DC biased ranging from 1 to 4 kV. The gate electrode was operated in pulse mode (10% duty ratio). Figure 1 shows a schematic diagram of the lateral-gate triode structure of the field emission device.

## Results and discussion

**Figure 2:**
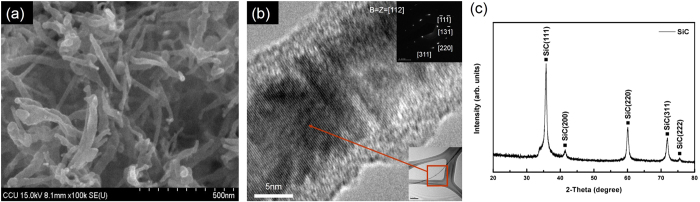
(**a**) SEM image of SiC nanowires, (**b**) TEM image of a single SiC nanowire(inset shows SAED pattern), and (**c**) XRD pattern of SiC nanowires.

**Figure 3:**
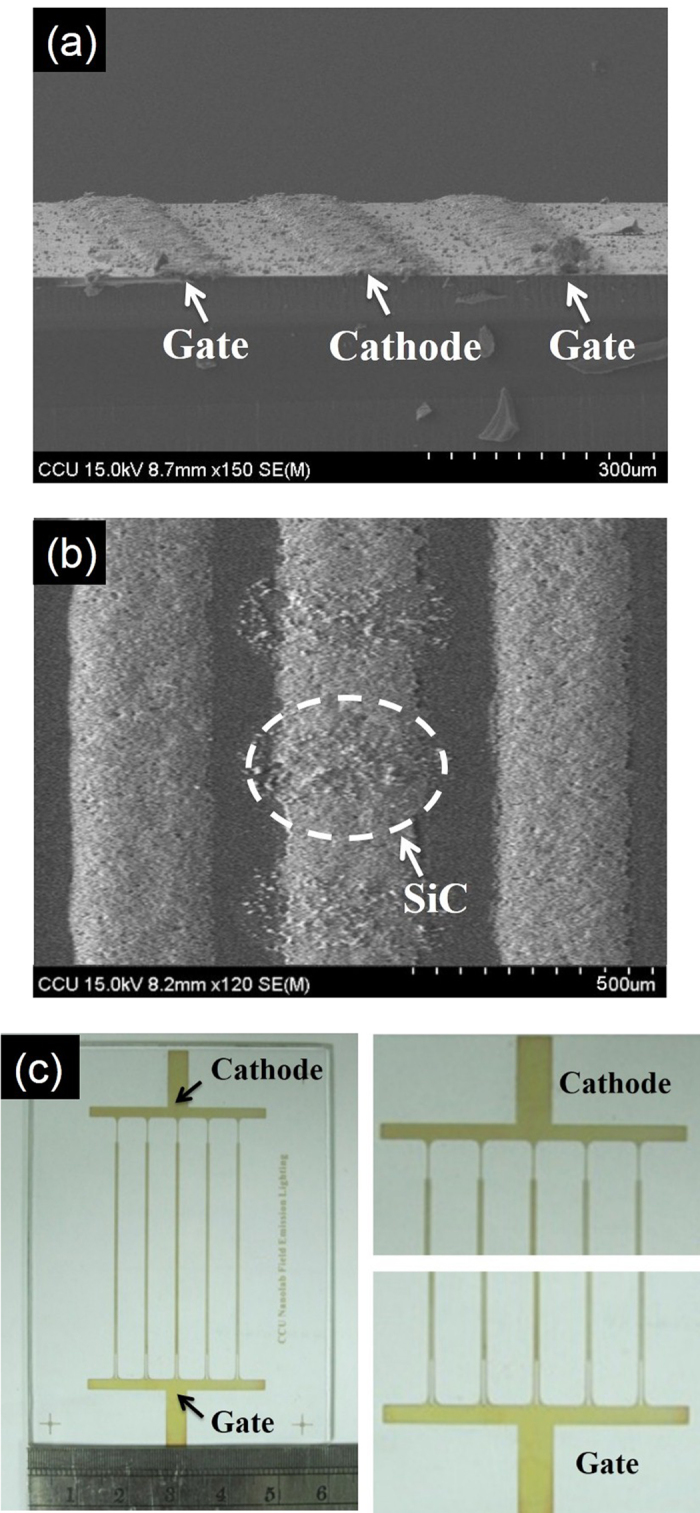
Lateral-gate structure of field emission device. (**a**)Cross-section and (**b**) top-view SEM images of a set of lateral-gate/cathode structure, and (**c**) panel with 5 sets of lateral-gate/cathode structure.

Figure 2(a) show SEM and TEM images and the XRD pattern of the produced SiC nanowires, respectively. The SiC nanowires have diameters in the range of 20–50 nm and lengths in the range of a few hundred nm to a few μm. A TEM image of a single SiC nanowire with a diameter of about 20–50 nm is shown in Fig. 2(b). The selected-area electron diffraction (SAED) pattern in the inset of Fig. 2(b) confirms that the nanowire has a single-crystalline β-SiC structure and that its growth direction was along the [111] plane. The XRD pattern of the SiC nanowires in Fig. 2(c) indicates that the material is the cubic structure of β-SiC. Figure 3(a) and (b) show cross-section and top-view SEM images of a lateral-gate/cathode structure, respectively. As can be seen in Fig. 3(a), three Ag electrodes were screen-printed with a height of 10 μm on the soda lime glass substrate. The two Ag electrodes on the sides act as the lateral-gate electrode and the Ag electrode in the center acts as the cathode. SiC nanowires as field emitters were screen-printed on the top of the center electrode. The top view of the structure in Fig. 3(b) shows that dot-like patterns consisted of SiC nanowires were screen-printed on the cathode electrode. The SiC nanowires were randomly dispersed within the dots and embedded in the center electrode. The diameter of the dots was about 150 μm. The distance between two adjacent dots was about 80 μm. The width of the electrodes was about 120 μm and the distance between two adjacent electrodes was about 80 μm. Figure 3(c) shows a panel with 5 sets of lateral-gate/cathode structure. The distance between two adjacent sets was about 6 mm. A soda lime glass substrate with an area of 6 × 8 cm^2^ and a thickness of 1.8 cm was used.

**Figure 4:**
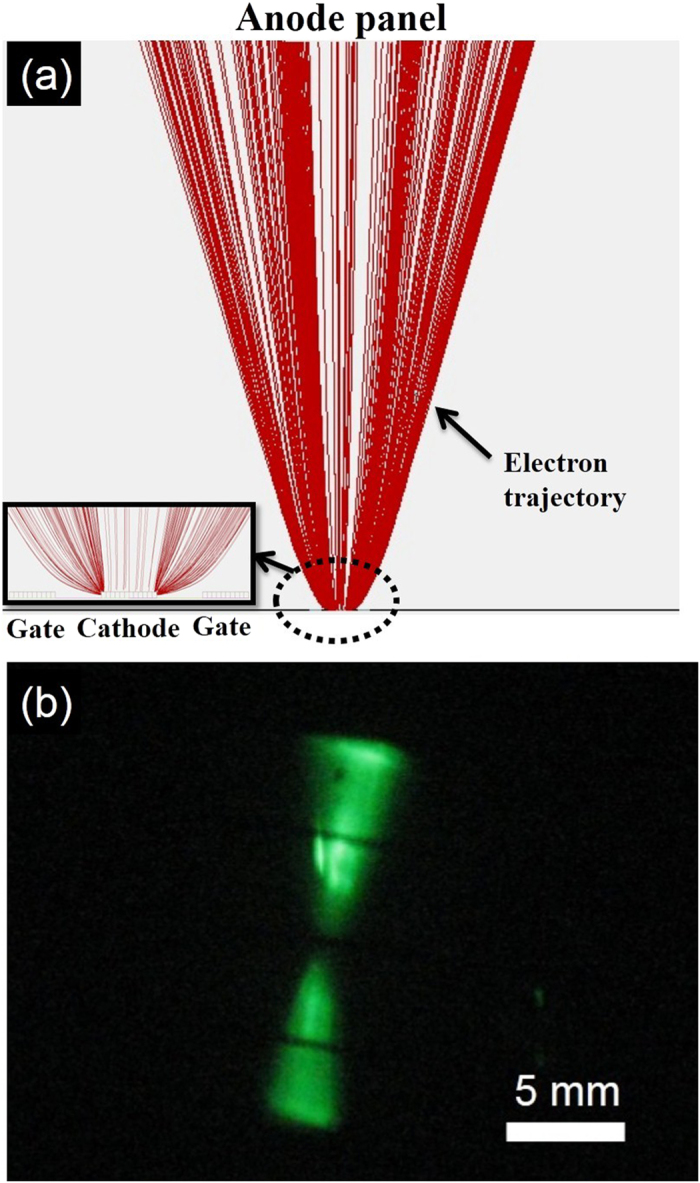
(**a**) Simulation and (**b**) experimental results of lateral-gate triode structure with a single Si nanowire dot on cathode.

**Figure 5:**
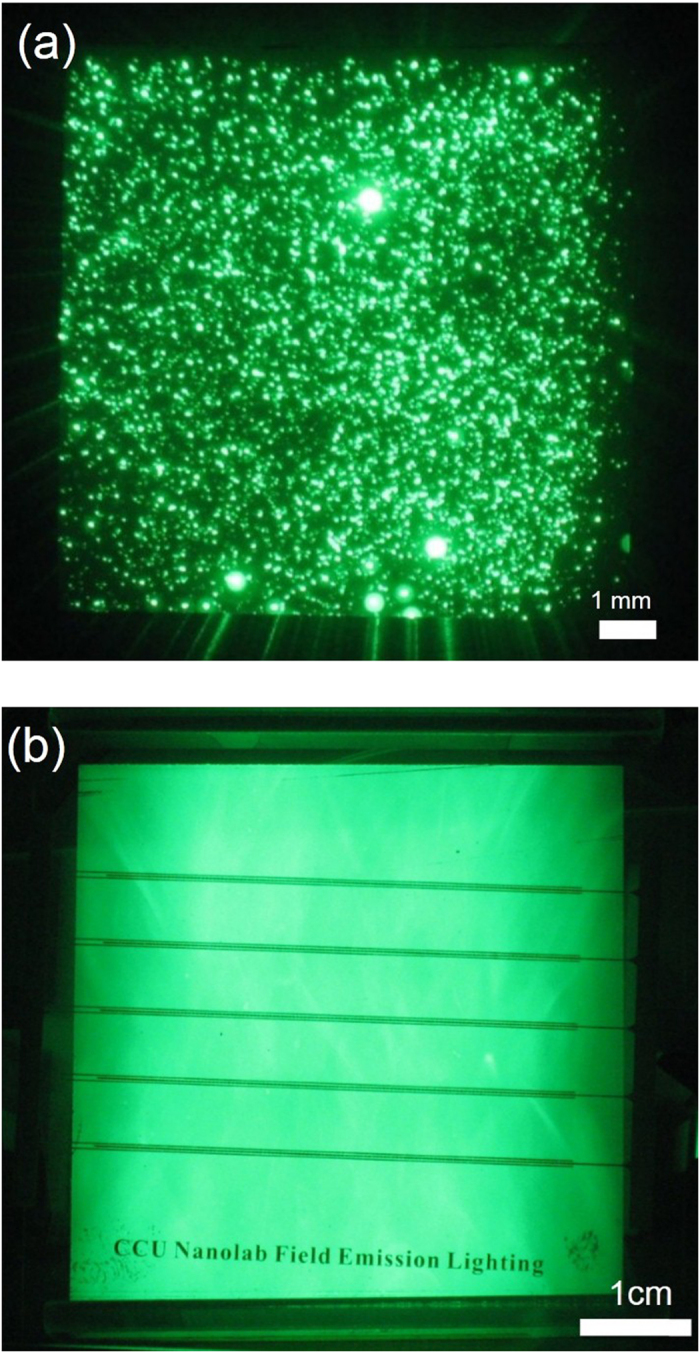
Luminance patterns produced by (**a**) conventional diode field emission structure and (**b**) lateral–gate triode structure.

Figure 4(a) and (b) show the simulation results obtained using SIMION software and the experimental results, respectively, for the lateral-gate triode structure with only a single dot on the cathode. The inset in Fig. 4(a) shows an enlarged image of the lateral-gate/cathode structure. The electrons were ejected from the emitters and moved toward the gate electrodes on both sides due to the quantum tunneling effect driven by the DC bias between the gate and cathode electrodes. Before the electrons reached the gate electrode, they changed direction and moved upward to the anode electrode due to the much higher DC bias between the cathode and anode electrodes. The electrons then reached the anode and bombarded the phosphors (if any), causing cathodoluminescence. In Fig. 4(a), the pattern of the electron trajectory shows that the electron beams were divergent, resulting in a large bombardment area on the anode. The divergent and uniformly distributed electron beams are beneficial for two-dimensional (2D) lighting applications. Figure 4(b) shows the top view of the luminance pattern in the anode for a single SiC dot. As expected, the experimental results well agree with the simulation results. The lateral-gate/cathode structure is thus capable of making divergent electron beams for 2D flat-panel lighting applications. This is different from the conventional triode structure, which makes convergent electron beams. Figure 5(a) and (b) show examples of luminance patterns of field emission for the conventional diode structure and the lateral-gate triode structure, respectively. As can be seen in Fig. 5(a), the phosphor screen (anode) shows a dot-like pattern. With increasing applied voltage between the cathode and the anode, the dots became brighter and more numerous. Eventually, the dots filled up the screen to become a 2D lighting panel. Poor brightness uniformity is obtained. In contrast, the lateral-gate structure produces a uniform and high-brightness luminance image, as shown in Fig. 5(b). No bright dots appear on the panel. The brightness can be controlled by changing the gate and anode voltages, allowing tunable luminance.

**Figure 6:**
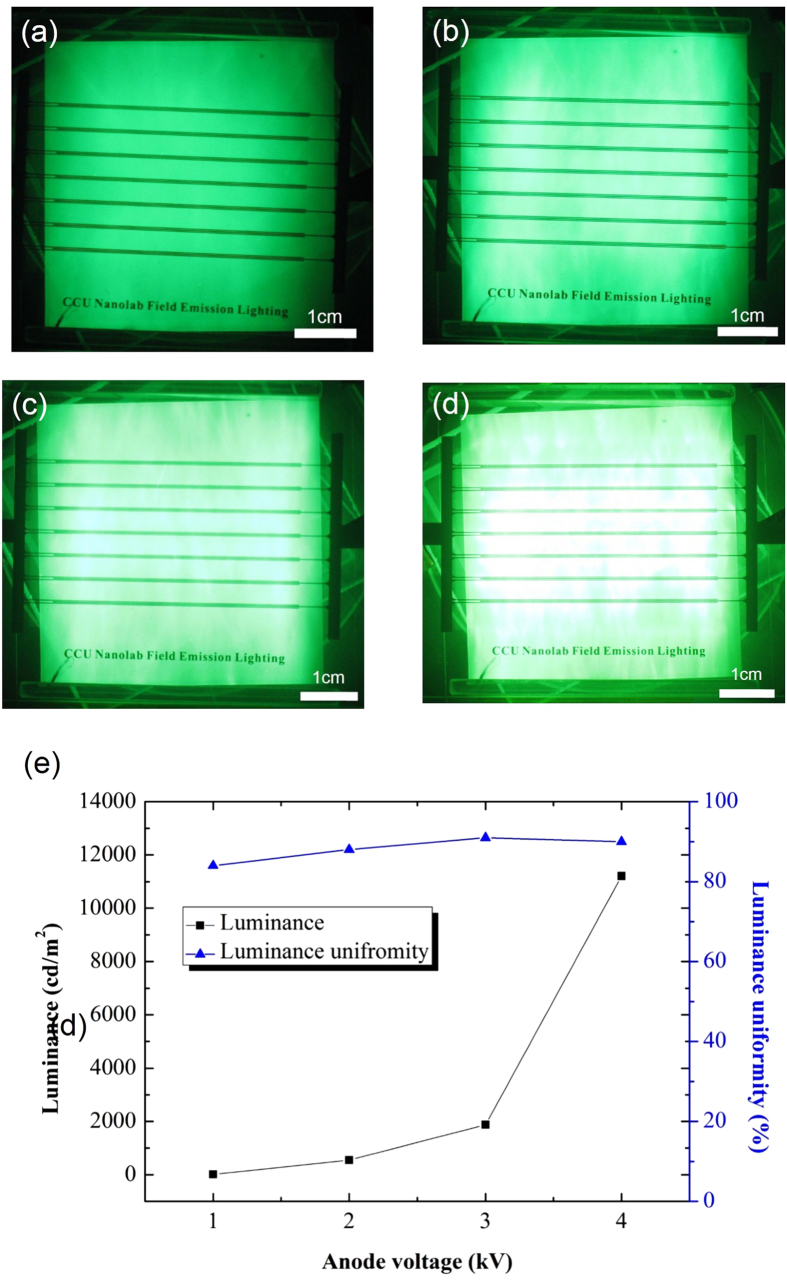
Luminance images of lateral-gate field emission device operating at anode voltages of (**a**) 1, (**b**) 2, (**c**) 3, and (**d**) 4 kV. (**e**) Luminance and luminance uniformity varying with anode voltage (gate voltage fixed at 500 V).

**Figure 7:**
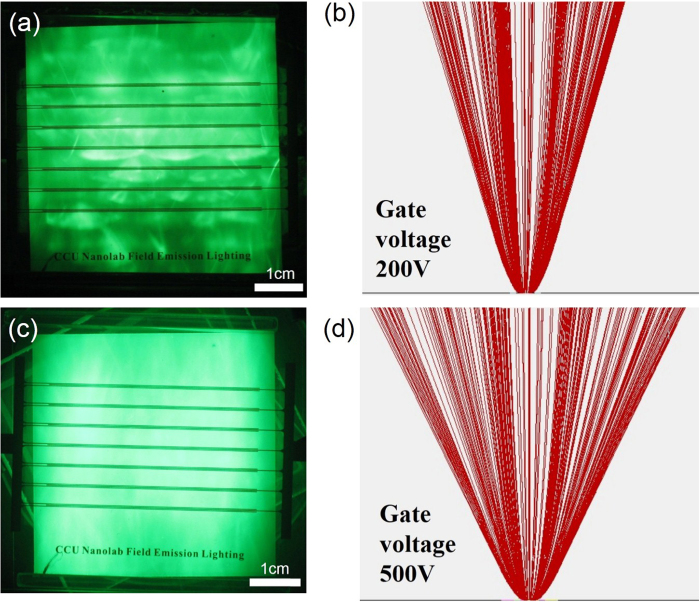
(**a**) Luminance image and (**b**) simulation results for lateral-gate structure with anode voltage of 2 kV and gate voltage of 200 V. (**c**) Luminance image and (**d**) simulation results for lateral-gate structure with anode voltage of 2 kV and gate voltage of 500 V.

Figure 6(a)–(d) show luminance images of the lateral-gate field emission device that uses a panel with 7 sets of lateral-gate/cathode structure, with cathode voltages of 1, 2, 3, and 4 kV, respectively. The gate voltage was kept at 500 V for all the tests. As can be seen, the luminance increased with increasing anode voltage. The luminance over the entire panel was quite uniform. The luminance uniformity was determined by following a 9-point procedure^19^. The 5 cm × 5 cm anode panel was divided into nine square areas for the brightness measurement. The luminance at the center point of each square was measured three times to obtain the average luminance. The luminance uniformity, *U*, can be expressed as:


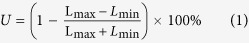


where *L*_*max*_ and *L*_*min*_ are the maximum and minimum luminance values measured at the nine points, respectively. In general, luminance uniformity is good when *U* ≧ 80%. The mean luminance of the entire panel was obtained by averaging the luminance values for the nine points. Figure 6(e) shows the mean luminance and luminance uniformity varying with the anode voltage. The luminance was 83 cd/m^2^ at an anode voltage of 1 kV, increasing slowly to 470 cd/m^2^ at 2 kV, and 2200 cd/m^2^ at 3 kV. The luminance then increased rapidly to 10952 cd/m^2^ when the anode voltage was 4 kV. The results suggest that the luminance can be tuned by adjusting the anode voltage. The luminance uniformity of the device was also very good. The uniformity values at 1, 2, 3, and 4 kV were 84.5%, 90%, 93%, and 90.5%, respectively. The kinetic energy of the emitted electrons from the cathode increased with increasing anode voltage. Electrons with a higher energy lead to higher luminance when they bombard the phosphors. Good luminance uniformity implies that the emitted electrons were well distributed on the anode due to the design of the lateral-gate structure and adequate operational conditions. The luminance of more than 10000 cd/m^2^ and the uniformity of higher than 90% meet the requirements for flat-panel lighting application. Experiments were conducted in which the gate voltage was varied to study luminance uniformity. Figure 7 shows the luminance images and simulation results for lateral-gate devices with gate voltages of 200 V (Fig. 7(a) and (c)) and 500 V (Fig. 7(b) and (d)), with the anode voltage kept at 2 kV. The results show that a poor uniformity of 75.7% was obtained for a gate voltage of 200 V, whereas a good uniformity of 90% was obtained for a gate voltage of 500 V, even though the two devices showed similar luminance (200 V: 456 cd/m^2^, 500 V: 470 cd/m^2^). Poor luminance uniformity is due to the insufficient gate voltage failing to “pull” electron beams wider toward the anode so that the coverage area of the electron beams in the anode was small. The narrow beam divergence caused the poor luminance uniformity. The simulation results shown in Fig. 7 (c) and (d) are in good agreement with the experimental results.

**Figure 8:**
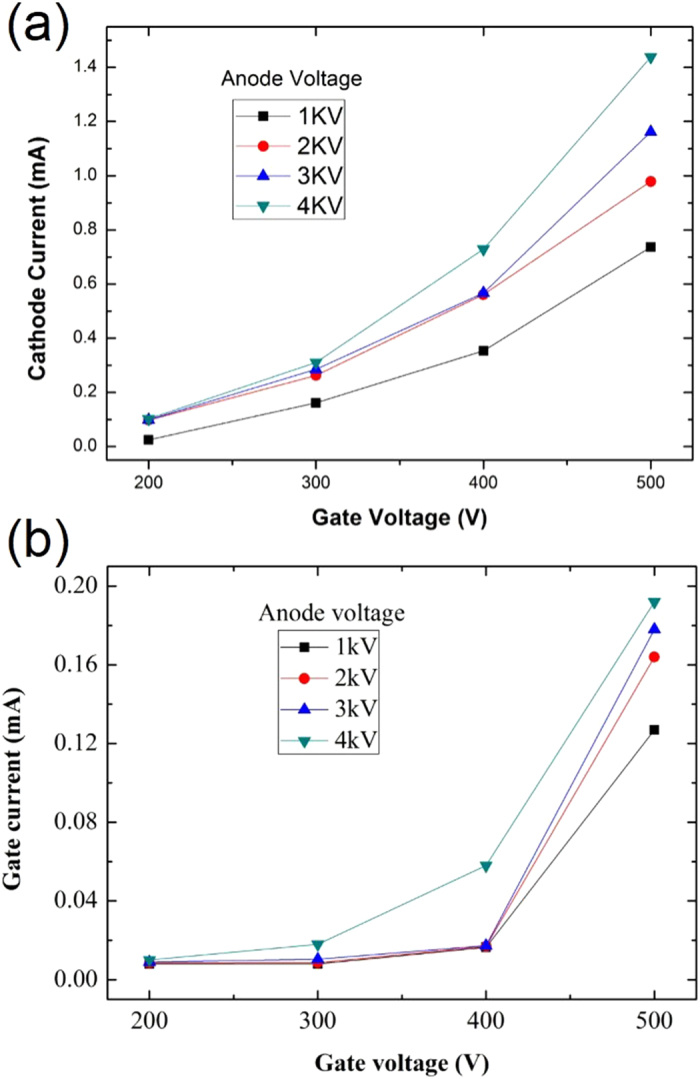
(**a**) Cathode and (**b**) gate currents varying with gate voltage and anode voltage.

Figure 8(a) and (b) show the cathode current and gate current, respectively, varying with gate and anode voltages. As can be seen in Fig. 8(a), the cathode current increased with increasing gate voltage. This is because the gate electrode acted as a field emission trigger for electrons to be emitted from the cathode. With increasing gate voltage, the electrical field near the SiC nanoemitters was enhanced so that a large number of the emitters were activated. High current was therefore induced in the anode due to the large number of electrons ejected from the emitters. For an anode voltage of 4 kV, cathode currents of 1.44 and 0.15 mA were obtained at gate voltages of 500 and 200 V, respectively. Figure 8(b) shows the gate current varying with gate and anode voltages. The gate current (leakage current) is an undesired current that commonly occurs in field emission devices with triode or tetrode structures. The current is caused by some emitted electrons impacting the gate before reaching the anode due to the bias voltage between the cathode and the gate. As expected, the gate current increased with increasing gate voltage, as shown in Fig. 8(b). For an anode voltage of 4 kV, the leakage currents were 0.019 and 0.010 mA (about 10% of the anode current) for gate voltages of 500 and 200 V, respectively.

**Figure 9:**
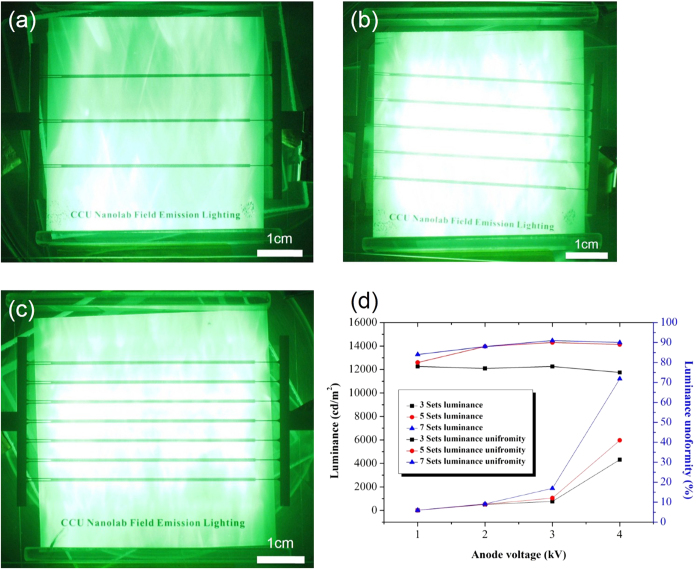
Luminance images for panels with (**a**) 3, (**b**) 5, and (**c**) 7 sets of lateral-gate/cathode structure, and (**d**) luminance and luminance uniformity varying with anode and gate voltages.

Figure 9 (a)–(c) show luminance images obtained using 3, 5, and 7 sets of the lateral-gate structure, respectively. An increase in the sets of the gate-cathode structure means an increase in the number of SiC nanoemitters in the panel, and thus an increase in the electron density per unit area (current density) for the bombardment of the phosphor anode. As a result, the luminance increased. Figure 9(d) shows the luminance and luminance uniformity varying with the number of sets of the lateral-gate/cathode structure. As expected, the luminance increased with increasing number of sets in the panel. The maximum luminance values were 4600, 5700, and 11000 cd/m^2^ for 3, 5, and 7 sets, respectively, with an anode voltage of 4 kV and a gate voltage of 500 V. The luminance uniformity was more than 90% for the devices with 5 and 7 sets but only 75–80% for the device with 3 sets. Although the novel lateral-gate structure enhanced the luminance uniformity dramatically, a 3-set structure was found to be insufficient.

## Conclusion

A field emission device with a lateral-gate triode structure was demonstrated. The results show that the structure improves luminance and luminance uniformity. The variations of luminance, luminance uniformity, anode current, and gate current with the gate voltage, anode voltage, and number of sets of gate-cathode electrodes were discussed. A simulation was conducted to confirm the luminance performance of the lateral-gate structure. A maximum luminance of 10952 cd/cm^2^ and a luminance uniformity of 93% can be achieved. In addition, the luminance can be tuned by varying the anode voltage.

## Additional Information

**How to cite this article**: Youh, M.-J. *et al.* Flat Panel Light Source with Lateral Gate Structure Based on SiC Nanowire Field Emitters. *Sci. Rep.* **5**, 10976; doi: 10.1038/srep10976 (2015).
